# Hormone replacement therapy, body mass, and the risk of colorectal cancer among postmenopausal women from Germany

**DOI:** 10.1038/sj.bjc.6604066

**Published:** 2007-11-06

**Authors:** M Hoffmeister, E Raum, J Winter, J Chang-Claude, H Brenner

**Affiliations:** 1 Division of Clinical Epidemiology and Aging Research, German Cancer Research Center, Heidelberg, Germany; 2 Department of General and Visceral Surgery, St Vincentius Hospital, Speyer, Germany; 3 Division of Cancer Epidemiology, German Cancer Research Center, Heidelberg, Germany

**Keywords:** colorectal cancer, body mass, postmenopausal, hormone replacement therapy, risk

## Abstract

Previous studies have reported inconsistent results regarding the modifying effect of hormone replacement therapy (HRT) on the association of body mass index (BMI) and the risk of colorectal cancer (CRC) among postmenopausal women. We assessed the use of HRT and BMI in 208 postmenopausal women with histologically confirmed incident CRC and 246 controls in a population-based case–control study in Germany (DACHS study). Ever use of HRT was strongly associated with reduction of CRC risk (adjusted odds ratio 0.41, 95% confidence interval 0.25–0.67). Among nonusers of HRT, risk of CRC was strongly increased in women with BMI 27 to <30 kg m^−2^ (2.76, 1.07–7.12) and obese women (3.30, 1.25–8.72), when compared with women with BMI <23 kg m^−2^ (*P* for trend <0.01). BMI was not associated with risk of CRC among HRT users (*P* for interaction <0.01). In contrast to most other studies, a positive association of BMI and CRC risk was found among nonusers of HRT, but not among users of HRT. The reasons for the inconsistency of results regarding the potential risk modifying effect of postmenopausal hormones in the association of BMI with CRC remain inconclusive and require further study.

Hormone replacement therapy (HRT) has been associated with risk reduction of colorectal cancer (CRC) in the Women's Health Initiative (WHI) Estrogen plus Progestin Study and a number of observational studies (Grodstein *et al*, 1999; Nanda *et al*, 1999; Chlebowski *et al*, 2004), while body mass index (BMI) ⩾25 kg m^−2^ was rather consistently associated with an increase of CRC risk in men and, less consistently and less pronounced, in women (Calle and Kaaks, 2004). Few studies have previously reported on the modifying effect of HRT on the association of BMI and CRC risk among postmenopausal women, and provided conflicting results (Table 1) (Slattery *et al*, 2003; Lin *et al*, 2004; Hou *et al*, 2006; Pischon *et al*, 2006; Adams *et al*, 2007; Wang *et al*, 2007). Thus, we investigated the association of postmenopausal hormone therapy and BMI with CRC risk in a large population-based study from Germany.

## MATERIALS AND METHODS

### Study design and study population

This investigation was conducted within the DACHS study, a population-based case–control study in the Rhine–Neckar–Odenwald region in Germany, which was primarily designed to assess the potential of endoscopic screening for the prevention of CRC. Details of the study design have been reported elsewhere (Brenner *et al*, 2006, 2007a, 2007b; Hoffmeister *et al*, 2007). Briefly, patients were included if invasive, and histologically confirmed CRC (ICD 10 pos. C18–C20) was diagnosed for the first time between January 2003 and June 2004. All 22 hospitals in the study region treating patients with CRC agreed to participate in the study. Control individuals were randomly selected from lists of population registries and frequency-matched by sex, 5-year age groups, and county of residence. Cases and controls were included if they were at least 30 years of age (no upper age limit), German speaking, and mentally and physically able to participate in a personal interview of about one hour. Control individuals with a history of CRC were excluded. If patients complied with the inclusion criteria, they were informed about the study by their treating physicians, mostly during hospital stay a few days after surgery. They were notified to the study centre upon receipt of informed consent. Controls were contacted by the study centre through mail and follow-up calls to ask for participation. All participants gave written informed consent. The study was approved by the Ethics Committees of the University of Heidelberg and the State Medical Boards of Baden–Wuerttemberg and Rhineland–Palatinate.

### Exposure assessment

Information on sociodemographic factors and a detailed medical and lifestyle history with regard to known or suspected protective factors or risk factors of CRC were collected by trained interviewers using a questionnaire. Risk factor information was collected for the time prior and up to the index date, which was the date of diagnosis for cases and the date of the interview for controls. In women, use of hormones was ascertained with regard to treatment of menopausal symptoms or for the prevention of diseases. Assessment of hormone use included reason, age at beginning and end of use (or current use), and total duration of use, but we had no information about the hormone preparations used. Only HRT use prior to diagnosis (cases) or interview (controls) was considered. We asked for current weight and previous weight at each decade of life (i.e., a person of 84 years was asked about current weight and weight at ages 80, 70, […], 20). To calculate BMI, we used the last available weight at least 5 years prior to the interview (range: 5–14 years, for a person of age 84 this would be weight at age 70). This measure of body weight was preferred over current BMI, because cancer-related symptoms before and after diagnosis, or therapy of CRC, might have caused weight changes.

Menopausal state at index date was defined by reported history of the women. Postmenopausal women were those whose menstrual bleedings had stopped naturally or after bilateral oophorectomy, radiation therapy, or chemotherapy, and all women older than age 55. However, menopausal state can be masked if hysterectomy was the reason for the cessation of the menstrual cycle, or if HRT was started before natural menopause (Beral, 2003). Thus, women ⩽55 years whose menstrual bleedings had stopped and, in addition, used HRT for the treatment of menopausal symptoms (a) in the past or (b) currently for more than 4 years (duration of perimenopause for most women (Dudley *et al*, 1998)) were also defined postmenopausal.

### Statistical analysis

We first compared the distribution of potential risk factors and protective factors among female postmenopausal cases and controls. Unconditional multiple logistic regression was employed to estimate odds ratios (ORs) for the association of HRT use and BMI with CRC risk. We included known or suspected risk factors or protective factors known from the literature and covariates with a significantly different distribution between cases and controls into the model. Then, a backward variable selection procedure was employed to eliminate all covariates that changed the OR of the exposure under investigation by 3% or less. In the final model, ORs were adjusted for the matching factors age (per year), county of residence, and other known or potential confounders: former colorectal endoscopy (yes/no), BMI (kg m^−2^), diagnosis of rheumatic disease (yes/no), diagnosis of hyperlipidaemia (yes/no, unknown), former participation in a general health screening examination (ever/never), ever regular use of nonsteroidal anti-inflammatory drugs (NSAIDs) including all aspirin use (at least two times per week for at least 1 year, yes/no), current regular use of statins (at least two times per week for at least 1 year, yes/no), average lifetime alcohol consumption (no use and tertiles among users in grams ethanol per day), oral contraceptive use (<3  years/ ⩾3 years), and lifetime pack-years of active smoking. Other potential confounders like first-degree family history of CRC, educational level, number of pregnancies longer than 6 months, induced menopause (bilateral oophorectomy, radiation therapy, or chemotherapy), lifetime physical activity, or frequent intake of red meat, fruits, and vegetables did not materially influence the estimates, and were not included in the model. Differences between cases and controls were evaluated using *χ*^2^-test, and multivariate analyses of linear trend were performed for duration of HRT use and along BMI categories. We tested for effect modification by introducing a multiplicative interaction term into the models. All analyses were carried out with the statistical software package SAS 9.1 (SAS Institute Inc., Cary, NC, USA).

## RESULTS

Overall, 540 patients and 614 control persons participated in the study (43% women). The patients recruited constitute about 50% of all eligible cases in the study region for the period of recruitment. Half of the interviews with cases were completed within 2 weeks after diagnosis, 75% within 1 month, and 88% within 6 months. Of all potentially eligible controls, 44% agreed to participate in the interview (another 25% provided less extensive information in a short questionnaire). Of the 492 women in the study, 34 pre- or perimenopausal women, and 4 women who provided no information about HRT use were excluded. The analysis was restricted to the remaining 208 patients and 246 controls.

The mean age of cases and controls was 69 and 70 years, respectively. Women with CRC had colon cancer in 68% and rectum cancer in 32% of cases (of which 5% were in the rectosigmoid), and tumour stages from I to IV were diagnosed in 15, 37, 28, and 20% of patients, respectively. The studied CRC patients and control subjects without CRC were not different regarding age, educational level, number of pregnancies for more than 6 months, removal of ovaries and uterus, unnatural induction of menopause by bilateral oophorectomy, chemotherapy, or radiation therapy, first-degree family history of CRC, alcohol consumption, or physical activity. Controls were more likely to have used oral contraceptives for at least 3 years, to use statins regularly, and to have smoked less than 30 pack-years of cigarettes. A statistically significant difference between cases and controls was found for BMI, former colorectal endoscopy, regular use of NSAIDs, and for a previous health check-up only (except for BMI, all more often controls, see Table 2).

Overall, HRT use was associated with strong reduction of CRC risk in postmenopausal women (OR 0.41, 0.25–0.67), mainly among current users and past users who had stopped HRT less than 5 years previously (Table 3). No evidence of interaction was found with previous colorectal endoscopy (*P*=0.52) or previous health check-up visits (*P*=0.18) as potential indicators of general health behaviour with HRT use. The association was similar for colon cancer (OR 0.35, 0.20–0.61) and for rectum cancer (0.54, 0.25–1.13). The mean duration of HRT use was 9.7, 14.2, and 6.2 years among ever, current, and past users, respectively. Although there was a significant trend (*P*<0.01), reduction of CRC risk did not appear to be stronger with duration of HRT use. The association of duration of HRT and reduction of CRC risk was similar among current and past users of HRT (data not shown).

Risk of CRC was increased in women with BMI 27 to <30 and 30+ kg m^−2^, when compared with women with BMI <23 kg m^−2^. Among nonusers of HRT, compared to those with BMI <23 kg m^−2^, women in higher BMI categories, 23 to <25, 25 to <27, 27 to <30, and 30+ kg m^−2^, were at increasing risk of CRC (*P*_trend_ <0.01). Odds ratios were statistically significant for BMI categories 27 to <30 and 30+ kg m^−2^. However, among women who ever used HRT, BMI was not associated with CRC risk (*P*_interaction_ <0.01) (Table 4).

## DISCUSSION

In this first large population-based case–control study from Germany, ever use of HRT was associated with strong and statistically significant reduction of CRC risk of 59%. However, only current or past use that ended less than 5 years previously was significantly associated with reduced risk of CRC. Risk reduction was already apparent after less than 5 years use (OR 0.38, 0.19–0.74) and did not seem to increase after 10–19 years or 20 years and longer. BMI was positively associated with increased risk of CRC among nonusers of HRT, but not among users of HRT.

Two previous randomised controlled trials (RCTs) on HRT investigated risk of CRC in postmenopausal women. The WHI was the first trial to report risk reduction of almost 40% by combination therapy of oestrogen plus progestin (Rossouw *et al*, 2002; Chlebowski *et al*, 2004), but not in women with previous hysterectomy using unopposed conjugated equine oestrogens (Anderson *et al*, 2004). CRC risk was, however, not reduced in postmenopausal women using oestrogen plus progestin in the HERS trial (hazard ratio (HR) 0.69, 0.32–1.49; Hulley *et al*, 2002).

In the meta-analysis by Grodstein *et al* (1999), four out of five studies providing information about the duration of HRT use found that protection was similar for all women currently taking hormones, regardless of duration (Calle *et al*, 1995; Folsom *et al*, 1995; Troisi *et al*, 1997; Grodstein *et al*, 1998; Paganini-Hill, 1999). Their summary relative risk for short-term use (RR 0.61, 0.48–0.79) was very similar to the relative risk for long-term use (RR 0.67, 0.56–0.79). In the WHI, a lower overall risk of CRC in the hormone group compared with the placebo group emerged after 4 years of treatment (La Vecchia *et al*, 2005). In the present study, risk reduction of long-term use ⩾10 years was comparable to that <5 years, even though a statistically significant trend towards stronger risk reduction with longer use was observed.

Previous studies have reported inconsistent results regarding the modifying effect of HRT on the association of body mass and risk of CRC among postmenopausal women (Table 1). In a large case–control study within a North American health maintenance organisation, Slattery *et al* (2003) reported that BMI was not related to CRC among postmenopausal women not using HRT, but among users of HRT: BMI from 23–30 to >30 kg m^−2^ was associated with two- to threefold risk of colon cancer, respectively, when compared with BMI <23 kg m^−2^. The mechanism by which oestrogens enhance the positive association was hypothesised to be upregulation of insulin growth factors, which leads to an increase in obesity-induced levels of insulin, and thereby increases CRC risk (Slattery *et al*, 2003). Lin *et al* (2004) analysed postmenopausal women of the Women's Health Study (WHS), a large RCT evaluating primary prevention of cancer and CVD by use of aspirin and vitamin E. In a cohort with a mean follow-up of 8.7 years, they found a positive association of BMI and CRC risk in both, current and never users of HRT. In another study within the large prospective NIH–AARP cohort, BMI was positively associated with colon cancer risk after 5 years follow-up among women aged 50–66 years only, and the association was not modified by HRT (Adams *et al*, 2007). On the other hand, in the EPIC study with a mean follow-up of 6.1 years, there was no association of BMI and risk of colon cancer among HRT users or nonusers, respectively (Pischon *et al*, 2006). One major difference between the WHS, the NIH–AARP, and the EPIC study compared with the study by Slattery *et al* (2003) was that the first three cohort studies defined current use of HRT at baseline (up to 8, 5, or 6 years prior to diagnosis), whereas in the latter case–control study, use of HRT was defined as use within the preceding 2 years. In a lean population from China with very rare HRT use, postmenopausal women in the highest BMI quintile (>23.6 kg m^−2^) had significantly lower risk of colon cancer than those with BMI<19 kg m^−2^. The authors hypothesised that higher levels of endogenous oestrogens increasing with BMI might outweigh the increased risk of CRC with higher BMI among women who are nonusers of HRT (Hou *et al*, 2006).

Yet another finding was obtained in the present study: among postmenopausal women who never used hormones, risk of CRC was increased in women with BMI 27 to <30 and 30+ kg m^−2^ two- to threefold that of women with BMI <23 kg m^−2^. A positive association of BMI and CRC risk was not found among HRT users (*P*<0.01). Recently, another large cohort study from the United States including 814 incident cases with CRC observed a statistically significant increase in CRC risk among nonusers of hormones with BMI⩾30 kg m^−2^ (HR 1.36, 1.04–1.79), but not among users, when compared to women with BMI<25 kg m^−2^ (Wang *et al*, 2007). However, despite the size of the study, no significant effect modification of the BMI–CRC association was found for never, former, and current use of HRT (*P*=0.5). Except for the WHS and the study by Wang *et al* (2007), all other previous studies differentiating between users and nonusers of HRT were mainly reporting on colon cancer risk (Slattery *et al*, 2003; Hou *et al*, 2006; Pischon *et al*, 2006; Adams *et al*, 2007), so we repeated the analysis after exclusion of patients with rectum cancer (32% of cases). Despite the limited power, ORs were similar for all BMI groups and risk of CRC was still significantly increased in the two highest BMI groups among those with no HRT use. Thus, our results rather indicate that use of HRT might neutralise the increase of CRC risk associated with overweight and obesity observed in previous studies (Bergström *et al*, 2001; Lin *et al*, 2004; Frezza *et al*, 2006). On the other hand, this would not be in line with the biologic mechanisms proposed by Slattery or Hou (Slattery *et al*, 2003; Hou *et al*, 2006). Furthermore, Pischon *et al* (2006) found that waist-to-hip ratio and waist circumference were positively associated with colon cancer risk among nonusers of HRT, but not among users of HRT, and suggested that measures of abdominal adiposity might be more closely linked to hormone effects than BMI.

The strengths of this study include a detailed assessment of exposure and covariable data. The DACHS study was conducted in a population-based setting, and lifetime exposures were assessed and analysed. In particular, we were able to include BMI in the years before diagnosis and HRT use up to the occurrence of CRC or the interview.

The study has also some limitations, which require further discussion. Despite follow-up by mail and phone, the rate of full participation among eligible control subjects in this study was slightly less than 50% in this population-based study requiring personal interviews, blood samples, access to medical records, and including cases and controls aged 75 or older who are more difficult to recruit. Only about 50% of eligible cases could be recruited. Patients were primarily missed due to work overload of the physicians in charge of recruitment in the hospitals, which is unlikely to be a source of selection bias. Only few patients refused to participate. Because of the very strict confidentiality rules in Germany, it was not possible to quantify these proportions precisely, as patients may only be contacted through the doctors in charge of their treatment. Information was available for self-reported start, end, total duration, and reason of HRT use from the interview, but may sometimes not have been adequate due to problems in recalling exact start or duration. However, previous validation studies have found that ever use of HRT and duration of use is well recalled (Goodman *et al*, 1990; Jain *et al*, 1999; Banks *et al*, 2001), and that recall in the present study is similar among cases and controls (Hoffmeister *et al*, 2007). If HRT was currently used, we only asked about the year of start and not the total duration of use. Interruption periods of several years and resumption of use, however, are not very likely to occur in users of HRT. The definition of menopausal state may not always be precise, because it was based on self-reported information of several factors. But most women in this study were older than 60 years or provided complete information for the definition of menopausal state. Body mass index was based on self-reported weight within 5–14 years prior to the index date and height. In previous validation studies, reported former weight has been shown to be accurate, but women were slightly more likely to underestimate their past weight, particularly those with higher BMI. However, nondifferential misclassification of BMI would rather have biased the estimates towards no association (Casey *et al*, 1991; Perry *et al*, 1995).

Women who chose to take postmenopausal hormones might differ from nonusers in ways that may affect CRC risk (Banks *et al*, 2002). For instance, many of the women in the present study were long-term users of HRT, which *per se* requires compliant behaviour that is generally associated with a healthier lifestyle and more physician contacts. However, major indicators of health behaviour or confounders that are associated with risk of CRC were ascertained and controlled for in the multivariate analysis, such as former health check-up visits or former endoscopy of the large intestine. Furthermore, there was no evidence of interaction for the effect of HRT use on CRC risk with previous endoscopy of the large intestine (*P*=0.52) or previous health check-up visits (*P*=0.18), respectively. However, the power of the study was limited to detect statistically significant interaction, and confounders might still have been measured inappropriately, or yet unknown confounders could have influenced this case–control study. Finally, we had no details about the hormone preparations prescribed. Potential differences in drug prescriptions, type of therapy and duration of use between Germany and other countries, particularly the United States, might have contributed to the inconsistency of results (Greiser, 2003; Löwel *et al*, 2003; Seifert-Klauss and Schumm-Draeger, 2003; Bromley *et al*, 2004; Hersh *et al*, 2004).

In conclusion, this first large population-based study of postmenopausal HRT and risk of CRC from Germany is compatible with existing evidence of a strong inverse relationship that was reported from other countries. Similar to earlier reports, risk reduction was apparent after less than 5 years of use, and did not decrease further with longer duration of use. A positive association of BMI and CRC risk was found among nonusers of HRT, but not among users of HRT, which raises the question whether risk reduction of CRC associated with HRT use might neutralise the increase in risk of CRC associated with increasing BMI. The reasons for the inconsistency of results regarding the role of postmenopausal hormones in the association of BMI with CRC risk remain inconclusive and require further study. In this regard, abdominal obesity might be more closely related to CRC than general body mass. Because risks seem to outweigh the benefits of hormone preparations studied in large RCTs, there seems to be no role for starting or continuing the prevention of CRC and other chronic diseases with HRT.

## Figures and Tables

**Table 1:** Previous studies reporting on the role of HRT in the association of BMI and risk of CRC among postmenopausal women

				**Relative risk (95% CI) among postmenopausal women**		
**First author, year**	**Study design and population**	**Assessment**	**BMI (kg m^−2^)**	**HRT use**	**No HRT use**	**Statistical interaction**
Slattery *et al* (2003)	Case–control study (USA), 734 postmenopausal colon cancer cases and 906 controls	HRT use within last 2 years, usual adult height and weight prior to diagnosis, colon cancer incidence	<23 23–24 25–27 28–30 >30	1.00 2.69 (1.34–5.40) 1.68 (0.83–3.38) 2.28 (1.07–4.87) 3.36 (1.58–7.13)	1.00 1.10 (0.73–1.66) 1.13 (0.79–1.62) 1.01 (0.68–1.51) 0.98 (0.67–1.44)	*P*<0.01
						
Lin *et al* (2004)	Cohort study (USA), 37 671 women (54% postmenopausal), 8.7 years follow-up, 155 postmenopausal women with CRC	BMI and HRT use at baseline, CRC incidence	<23 23.0–24.9 25.0–26.9 27.0–29.9 ⩾30	1.00 0.81 (0.36–1.84) 1.08 (0.47–2.44) 1.98 (0.98–3.99) 1.41 (0.65–3.06)	1.00 2.00 (0.98–4.10) 1.32 (0.58–3.02) 1.05 (0.42–2.65) 2.91 (1.40–6.06)	*P*=0.33
						
Pischon *et al* (2006)	Cohort study (Europe), 368 277 women and men, 6.1 years follow-up, 424 postmenopausal women with colon cancer	BMI and HRT use at baseline, colon cancer incidence	<21.7 21.7–23.5 23.6–25.7 25.8–28.8 ⩾28.9	1.00 0.69 (0.35–1.35) 0.80 (0.41–1.56) 1.10 (0.57–2.10) 0.72 (0.31–1.70)	1.00 0.96 (0.63–1.45) 1.21 (0.82–1.78) 1.11 (0.75–1.64) 1.12 (0.75–1.67)	Not reported
						
Hou *et al* (2006)	Case–control study (China), 317 postmenopausal colon cancer cases and 534 controls	Usual adult weight and height, population with very rare HRT use (1%), colon cancer incidence	⩽19.0 19.1–20.5 20.6–21.9 22.0–23.6 >23.6	— — — — —	1.00 1.1 (0.6–1.5) 0.8 (0.5–1.2) 0.8 (0.6–1.4) 0.6 (0.3–0.9)	Not applicable
						
Adams *et al* (2007)	Cohort study (USA), 517 144 women and men, ∼5 years follow-up, 758 postmenopausal women with CRC	BMI and HRT use at baseline (age 50–71), colon cancer incidence	18.5 to <23 23 to <25 25 to <27.5 27.5 to <30 30 to <35 ⩾35	1.00 1.07 (0.70–1.63) 1.56 (1.06–2.29) 1.52 (0.96–2.40) 1.69 (1.09–2.64) 1.55 (0.85–2.84)	1.00 1.41 (1.04–1.92) 1.29 (0.96–1.75) 1.26 (0.90–1.77) 1.05 (0.75–1.48) 1.38 (0.96–1.99)	*P*=0.28
Wang *et al* (2007)	Cohort study (USA), 97 787 women, ∼10 years follow-up, 814 postmenopausal women with CRC	BMI 10 years before baseline, HRT assessed three times during follow-up, CRC incidence	18.5–24.9 25.0–29.9 ⩾30 18.5–24.9 25.0–29.9 ⩾30	*Former use* 1.00 1.13 (0.85–1.52) 0.92 (0.61–1.39) *Current use* 1.00 1.09 (0.80–1.47) 1.14 (0.74–1.75)	1.00 1.08 (0.85–1.37) 1.36 (1.04–1.79)	*P*=0.5

BMI=body mass index; CI=confidence interval; CRC=colorectal cancer; HRT=hormone replacement therapy.

**Table 2:** Characteristics of the study population

	**Cases *N*=208**	**Controls *N*=246**	***χ*^2^-test**
*Age at index year (years)*			
40–49	2 (1%)	1 (<1%)	
50–59	24 (12%)	34 (14%)	
60–69	73 (35%)	101 (41%)	
70–79	67 (32%)	80 (33%)	
80+	42 (20%)	30 (12%)	*P*=0.16
			
*BMI (kg m*^−*2*^)^a^			
<23.0	51 (25%)	73 (30%)	
23 to <25	39 (19%)	48 (20%)	
25 to <27	25 (12%)	49 (20%)	
27 to <29	46 (23%)	36 (15%)	
30+	40 (20%)	38 (16%)	*P*=0.04
			
*Educational level*			
Low	153 (74%)	181 (74%)	
Intermediate	36 (17%)	41 (17%)	
High	19 (9%)	21 (10%)	*P*=0.96
			
*Pregnancies* >*6 months*			
0	34 (16%)	32 (13%)	
1	58 (28%)	55 (22%)	
2	69 (33%)	99 (40%)	
3+	47 (23%)	60 (24%)	*P*=0.27
			
*Oral contraceptive use* b			
<3 years	146 (72%)	157 (64%)	
⩾3 years	58 (28%)	87 (36%)	*P*=0.10
			
Bilateral oophorectomy	26 (12%)	27 (11%)	*P*=0.61
			
Hysterectomy^c^	69 (33%)	89 (36%)	*P*=0.53
			
Induced menopause	14 (7%)	16 (6%)	*P*=0.92
			
First-degree family history of CRC^d^	31 (15%)	34 (14%)	*P*=0.71
			
Former colorectal endoscopy^e^	42 (20%)	116 (47%)	*P*<0.01
			
Regular use of NSAIDs^f^	57 (27%)	101 (41%)	*P*<0.01
Regular use of statins^g^	16 (8%)	32 (13%)	*P*=0.06
			
			
Former health check-up^h^	148 (72%)	212 (87%)	*P*<0.01
			
*Active smoking (lifetime pack-years)* i			
Never active	140 (67%)	168 (69%)	
<10	26 (12%)	37 (15%)	
10–19	15 (7%)	19 (8%)	
20–29	9 (4%)	14 (6%)	
30+	18 (9%)	7 (3%)	*P*=0.10
			
*Alcohol, nonuser, and tertiles among users (average lifetime ethanol, g day*^−*1*^)^j^			
0	74 (36%)	71 (29%)	
0.1–4.1	44 (21%)	59 (24%)	
4.1–9.3	43 (21%)	60 (24%)	
>9.3	46 (22%)	56 (23%)	*P*=0.44
*Physical activity, quartiles (average lifetime METs, h week*^−*1*^)^k^			
⩽150.3	57 (28%)	56 (23%)	
150.4–220.4	49 (24%)	63 (26%)	
220.4–297.9	48 (24%)	65 (26%)	
>297.9	50 (25%)	62 (25%)	*P*=0.64

BMI=body mass index; CRC=colorectal cancer; NSAID=nonsteroidal anti-inflammatory drug.

a BMI 5–14 years before interview, data missing for seven cases and two controls.

b Data missing for four cases and two controls.

c Data missing for one case.

d Data missing for two cases.

e Data missing for one case.

f Ever regular use of NSAIDs including aspirin (2+ times per week for >1 year).

g Current use; data missing for four controls.

h Data missing for three cases and one control.

i Data missing for one control.

j Data missing for one case.

k Data missing for four cases.

**Table 3:** Association of HRT use with the risk of CRC

			**OR (95% CI)^a^**	
	**Cases**	**Controls**	**Basic model^b^**	**Adjusted modelb,^c^**
No HRT use	144 (69%)	104 (42%)	1.00 Ref	1.00 Ref
*HRT use ever*	64 (31%)	142 (58%)	0.30 (0.19–0.46)	0.41 (0.25–0.67)
				
Current use	29 (14%)	61 (26%)	0.29 (0.17–0.52)	0.35 (0.19–0.67)
Past use^d^	35 (17%)	81 (32%)	0.31 (0.19–0.51)	0.45 (0.25–0.79)
				
Last use 1–4 years previously	11 (5%)	32 (14%)	0.21 (0.10–0.45)	0.26 (0.11–0.63)
Last use 5–9 years previously	8 (4%)	12 (5%)	0.47 (0.18–1.23)	0.68 (0.24–1.97)
Last use ⩾10 years previously	12 (6%)	26 (11%)	0.34 (0.16–0.71)	0.64 (0.24–1.50)
				
Duration of HRT use				
<5 years	19 (9%)	54 (22%)	0.24 (0.13–0.45)	0.38 (0.19–0.74)
5–9 years	16 (8%)	21 (9%)	0.51 (0.24–1.07)	0.70 (0.31–1.60)
10–19 years	22 (11%)	48 (20%)	0.29 (0.16–0.54)	0.36 (0.18–0.71)
20+ years	7 (3%)	19 (8%)	0.25 (0.10–0.62)	0.34 (0.13–0.92)
*P* for trend^e^			P<0.01	P<0.01

BMI=body mass index; CI=confidence interval; CRC=colorectal cancer; HRT=hormone replacement therapy; NSAID=nonsteroidal anti-inflammatory drug; OR=odds ratio.

a Effects of HRT are implicitly weighted across categories of BMI 5–14 years ago.

b ORs adjusted for matching factors only (age and county of residence).

c ORs additionally adjusted for BMI, history of rheumatic disease, hyperlipidaemia, former health check-up, former colorectal endoscopy, pack-years of smoking, alcohol, regular use of NSAIDs, regular use of statins, and oral contraceptive use.

d Year of last use of HRT missing for 11 cases and 4 controls.

e Test statistic was calculated for the following categories: no use, <5 years, 5–9 years, 10–19 years, and 20+ years.

**Table 4:** Association of BMI and risk of CRC among postmenopausal women

			**OR (95% CI)**	
**BMI, 5–14 years ago (kg m^−2^)^a^**	**Cases**	**Controls**	**Basic model^b^**	**Adjusted modelb,^c^**
*All women* d				
<23	51 (25%)	73 (30%)	1.00 Ref	1.00 Ref
23 to <25	39 (19%)	48 (20%)	1.19 (0.67–2.11)	0.80 (0.42–1.53)
25 to <27	25 (12%)	49 (20%)	0.72 (0.39–1.34)	0.78 (0.39–1.58)
27 to <30	46 (23%)	36 (15%)	1.80 (1.01–3.20)	1.71 (0.89–3.31)
30+	40 (20%)	38 (16%)	1.60 (0.89–2.87)	1.82 (0.92–3.62)
*P* for trend			*P*=0.05	0.02
				
*Never use of HRT*				
<23	24 (18%)	27 (26%)	1.00 Ref	1.00 Ref
23 to <25	31 (23%)	25 (24%)	1.45 (0.66–3.17)	1.31 (0.55–3.12)
25 to <27	18 (13%)	18 (17%)	1.18 (0.49–2.84)	1.60 (0.58–4.44)
27 to <30	33 (24%)	16 (16%)	2.19 (0.94–5.09)	2.76 (1.07–7.12)
30+	31 (23%)	17 (17%)	2.11 (0.91–4.88)	3.30 (1.25–8.72)
*P* for trend			*P*=0.05	*P*<0.01
				
*Ever use of HRT*				
<23	27 (42%)	46 (33%)	1.00 Ref	1.00 Ref
23 to <25	8 (13%)	23 (16%)	0.57 (0.21–1.55)	0.49 (0.16–1.48)
25 to <27	7 (11%)	31 (22%)	0.38 (0.14–1.01)	0.36 (0.11–1.13)
27 to <30	13 (20%)	20 (14%)	1.37 (0.55–3.41)	1.18 (0.40–3.48)
30+	9 (14%)	21 (15%)	0.68 (0.25–1.81)	0.89 (0.29–2.75)
*P* for trend			*P*=0.75	*P*=0.96

BMI=body mass index; CI=confidence interval; CRC=colorectal cancer; HRT=hormone replacement therapy; NSAID=nonsteroidal anti-inflammatory drug; OR=odds ratio.

a BMI missing for seven cases and two controls.

b ORs adjusted for matching factors only (age and county of residence).

c ORs additionally adjusted for BMI, history of rheumatic disease, hyperlipidaemia, former health check-up, former colorectal endoscopy, pack-years of smoking, alcohol, regular use of NSAIDs, regular use of statins, and oral contraceptive use.

d Additional adjustment for hormone replacement therapy.
